# Flow and Heat Transfer to Sisko Nanofluid over a Nonlinear Stretching Sheet

**DOI:** 10.1371/journal.pone.0125683

**Published:** 2015-05-18

**Authors:** Masood Khan, Rabia Malik, Asif Munir, Waqar Azeem Khan

**Affiliations:** Department of Mathematics, Quaid-i-Azam University, Islamabad 44000, Pakistan; North China Electric Power University, CHINA

## Abstract

The two-dimensional boundary layer flow and heat transfer to Sisko nanofluid over a non-linearly stretching sheet is scrutinized in the concerned study. Our nanofluid model incorporates the influences of the thermophoresis and Brownian motion. The convective boundary conditions are taken into account. Implementation of suitable transformations agreeing with the boundary conditions result in reduction of the governing equations of motion, energy and concentration into non-linear ordinary differential equations. These coupled non-linear ordinary differential equations are solved analytically by using the homotopy analysis method (HAM) and numerically by the shooting technique. The effects of the thermophoresis and Brownian motion parameters on the temperature and concentration fields are analyzed and graphically presented. The secured results make it clear that the temperature distribution is an increasing function of the thermophoresis and Brownian motion parameters and concentration distribution increases with the thermophoresis parameter but decreases with the Brownian motion parameter. To see the validity of the present work, we made a comparison with the numerical results as well as previously published work with an outstanding compatibility.

## Introduction

Fluid heating/cooling is essential in diverse fields such as power manufacturing and transportation. Viable heating/cooling strategies are completely required for cooling of any kind of high energy device. There are a few routines that enhance the heat transfer effectively. Few systems used extended surfaces, utilization of micro-channels and application of vibration to the heat transfer surfaces. Heat transfer proficiency can additionally be enhanced by improving the base fluid's thermal conductivity [1].

Owing to the low heat transfer properties, the fluids utilized for the purpose of heat transfer in general for example, ethylene glycol, water, glycerol and motor oils have restricted heat transfer abilities. However, metal and metal oxide such as metals (Cu, Ag, Au), carbon oxide ceramics (Al_2_O_3_, CuO), carbide ceramics (Sic, Tic) and metal nitrides (AlN, SiN) etc. nanoparticles have distinctive physical and chemical properties [2] and having thermal conductivities considerably higher than the liquids. Consequently, the concept of introducing particles in an ordinary fluid to provide a better heat transfer medium that acts like a liquid possessing metal's thermal conductivity was emerged. Amongst all the dimensions of added particles (such as macro, micro and nano) the nano-scaled particles have attracted more consideration by researchers being the fact of keeping the mixture homogeneous. The unique quality of nanofluids is thermal conductivity improvement, a perception noticed by Masuda *et al*. [3]. Nanofluids are imagined to depict liquids in which nanometer-sized particles (in general <100 nm in size) are suspended in convectional heat transfer base fluids. Nanofluids are vital in various industrial sectors including transportation, chemical and metallurgical sectors, micro manufacturing, power generation, thermal therapy for the treatment of cancer and, micro manufacturing and so forth. For the reason of their brilliant combined wetting and diffusing nature, nanofluids are also essential for the production of nanostructured materials, for the building of complex fluids and for the refining oil from surfaces and so forth.

In the interim, hypothetical studies were carried out to model nanofluids behavior. Macroscopic models for nanofluid flow and heat transfer can be classified as single-phase and two-phase models. Single-phase approaches consider nanoparticles and base fluid as a single homogeneous fluid with respect to its effective properties. Two-phase approaches handle continuity, momentum and energy equations for particles and base fluid using three different methods. Although two-phase models provide a better understanding of both phases, single-phase models are computationally more efficient, however provide less detail about each phase.

Buongiorno [4] observed that homogeneous models have a tendency to under estimate the nanofluid heat transfer coefficient and because of nanoparticle size, the dispersion impact is entirely insignificant. Later on, Buongiorno [4] proposed a model to clarify the irregular convective heat transfer enhancement in nanofluids and removed the deficiencies of the homogenous and dispersion models. He considered seven slip systems, including inertia, Brownian diffusion, thermophoresis, diffusiophoresis, Magnus, fluid drainage and gravity. He affirmed that only Brownian diffusion and thermophoresis are dominant slip mechanisms in nanofluids. Further, Buongiorno [4] reasoned that turbulence is not influenced by nanoparticles. In light of this discovery, he suggested a four-equations non-homogeneous two-component equilibrium model for convective transport in nanofluids. Consequently, within the recent years, the use of nanofluids to improve heat transfer has drawn respectable attention amongst researchers.

The problems involving boundary-layer flow of nanofluids have been studied by several authors. For the sake of brevity, we mention here some examples. Khan and Pop [5] scrutinized the flow, heat transfer and nanoparticle volume fraction towards a linearly stretched surface in a nanofluid. Rahman *et al*. [6] numerically analyzed the flow and heat transfer characteristics of nanofluids over an exponentially shrinking/stretching surface. They utilized Buongiorno's model with second order slip velocity and obtained dual solutions for the shrinking (*λ* <0) and stretching (*λ* >0) cases. Unsteady flow past a continuously shrinking surface with wall mass suction in the nanofluid is examined by Rohni *et al*. [7]. They found the dual results for specific values of wall mass suction, unsteadiness and nanofluid parameters. Rosca and Pop [8] investigated unsteady flow and heat transfer of a nanofluid in an external uniform free stream towards a moving surface. They discussed the stability analysis and noted that upper branch solutions are stable, while the lower branch solutions are not. Unsteady flow and heat transfer of a nanofluid past a contracting cylinder is studied by Zaimi *et al*. [9]. Malvandi and Ganji [10] provided a theoretical investigation of an MHD flow and heat transfer of nanofluid inside an isothermal circular microchannel. They considered Navier's slip boundary condition on the walls. Khan and Aziz [11] considered the Buongiorno's model to examine the double-diffusive natural convection from the vertical plate to the porous space saturated with a base fluid containing nanoparticles. In their work Hady *et al*. [12] studied the flow and heat transfer characteristics of a viscous nanofluid over a nonlinearly stretching sheet when incorporating the thermal radiation effects in the energy equation. Also, nanofluid flow with yield stress effect was analyzed by Hady *et al*. [13] over a sheet stretching nonlinearly in a porous medium.

To face the challenges in the area of science and technology researchers are compelled to study the non-Newtonian fluids, as they occur more frequently in real life applications. The non-Newtonian fluids are more extensive class of fluids which can't be depicted by a solitary model because the ability of each model for portraying the fluid properties is limited. The Sisko fluid model [14] is one of the most important fluid models portraying the flow in the power-law and upper Newtonian regions. Aforementioned model is most appropriate to describe the flow behavior of fluids in high shear rate regions. Initially this model was introduced to describe the flow of greases as, greases have high viscosities at low shear rates and low viscosities at high shear rates, but it was found later that it also describes the flow behavior of cement pastes etc. Its industrial applications incorporate drilling fluids, cement slurries and waterborne coatings etc.

It appears from the literature survey that no exploration has been done on the boundary layer flow and heat transfer to Sisko nanofluid towards a nonlinearly stretching flat plate in the presence of convective boundary conditions. Therefore, our present study aimed to explore the local-similarity solutions [15] of the transformed nonlinear coupled ordinary differential equations for diverse estimations of the controlling parameters. The model introduced by Buongiorno has been used in the present study. The effects of the controlling parameters on the dimensionless velocity, temperature, nanoparticles volume fraction, the rate of heat transfer and the rate of a nanoparticles volume fraction will be presented to gain thorough insight towards the physics of the problem.

## Problem Formulation

Consider the laminar, two-dimensional, steady flow and heat transfer of the Sisko nanofluid in the region *y* > 0 driven by a sheet stretching with power-law velocity *U* = *cx* ^*s*^, where *c* represents a non-negative real number and *s* > 0 represents the stretching rate of the sheet. The stretching sheet is assumed to be coinciding with the *x* − axis while the *y* − axis is perpendicular to the plane of the sheet. A hot fluid with temperature *T*_*f*_ is utilized to heat up or cool down the surface of the sheet (to be determined later) by convective heat transfer mode, which provides the heat transfer coefficient *h*_*f*_. We assume the uniform nanoparticle volume fraction of the surface of the stretching sheet is *C*_*w*_, whereas the ambient temperature and nanoparticle volume fraction are *T*_*∞*_ and *C*_*∞*_, respectively. Under these assumptions along with boundary layer approximation the system of equations which governs the forced convective boundary-layer flow is given by

$$\frac{\partial u}{\partial x}+\frac{\partial v}{\partial y}=0,$$ (1)

$$u\frac{\partial u}{\partial x}+v\frac{\partial u}{\partial y}=\frac{a}{\rho }\frac{\partial^{2}u}{\partial y^{2}}-\frac{b}{\rho }\frac{\partial }{\partial y}{\left(-\frac{\partial u}{\partial y}\right)}^{n},$$ (2)

$$u\frac{\partial T}{\partial x}+v\frac{\partial T}{\partial y}=\alpha \frac{\partial^{2}T}{\partial y^{2}}+\tau \left[D_{B}\frac{\partial C}{\partial y}\frac{\partial T}{\partial y}+\frac{D_{T}}{T_{\infty }}{\left(\frac{\partial T}{\partial y}\right)}^{2}\right],$$ (3)

$$u\frac{\partial C}{\partial x}+v\frac{\partial C}{\partial y}=D_{B}\frac{\partial^{2}C}{\partial y^{2}}+\frac{D_{T}}{T_{\infty }}\frac{\partial^{2}T}{\partial y^{2}}.$$ (4)

The associated boundary conditions are as follows:

u(x,y)=U=cxs,v(x,y)=0,k∂T(x,y)∂y=−hf[Tf−T(x,y)],C=Cwaty=0,u→0,v→0,T→T∞,C→C∞asy→∞. (5)

Here *u* and *v* denote the components of velocity along *x* − and *y* − axes respectively, *a*, *b* and *n* (*n* ≥ 0) are the material constants of the Sisko fluid, *T* and *C* represent the temperature and solid nanoparticle volume fraction, *ρ*, *σ*, *α* (= *k* / (*ρc*_*p*_)_*f*_) and *k* represent the fluid density, electrical conductivity, thermal diffusivity and thermal conductivity. Furthermore, *τ* (= (*ρc*)_*p*_ / (*ρc*)_*f*_) represents the ratio of effective heat capacity of the nanoparticle material (i.e., (*ρc*)_*p*_) to the heat capacity of the fluid (i.e., (*ρc*)_*f*_), *D*_*B*_ and *D*_*T*_ represent the Brownian diffusion coefficient and thermophoresis diffusion coefficient, respectively.

The dimensionless velocity, temperature and nanoparticle volume fraction are given as:

$$f\prime =\frac{u}{U},\;\theta =\frac{T-T_{\infty }}{T_{f}-T_{\infty }},\;\varphi =\frac{C-C_{\infty }}{C_{w}-C_{\infty }}.$$ (6)

In perspective of the above non-dimensional variables, we obtain

$$\begin{array}{l} u\left(x,\;y\right)=Uf\prime \left(\eta \right),\;\eta =\frac{y}{x}{\text{Re}}_{b}^{\frac{1}{n+1}},\\ v\left(x,\;y\right)=-U{\text{Re}}_{b}^{-\frac{1}{n+1}}\frac{1}{n+1}\left[\left\{s\left(2n-1\right)+1\right\}\;f(\eta )+\left\{s\left(2-n\right)-1\right\}\;\eta f\prime \left(\eta \right)\right]. \end{array}$$ (7)

In view of Eqs (6) and (7), the above governing problem can be written as

$$Af\prime \prime \prime +n{\left(-f\prime \prime \right)}^{n-1}\;f\prime \prime \prime +\left(\frac{s\left(2n-1\right)+1}{n+1}\right)\;ff\prime \prime -s{\left(f\prime \right)}^{2}=0,$$ (8)

$$\theta \prime \prime +\text{Pr}\left(\frac{s\left(2n-1\right)+1}{n+1}\right)f\;\theta \prime +N_{b}\varphi \prime \theta \prime +N_{t}\theta^{\prime 2}=0,$$ (9)

$$\varphi \prime \prime +\text{Pr}\;Le\left(\frac{s\left(2n-1\right)+1}{n+1}\right)f\varphi \prime +\frac{N_{t}}{N_{b}}\theta \prime \prime =0,$$ (10)

$$\begin{array}{l} \;f\left(0\right)=0,\;f\prime \left(0\right)=1,\;\theta \prime (0)=-\gamma \left[1-\theta \left(0\right)\right],\;\varphi \left(0\right)=1,\\ f\prime \left(\infty \right)\to 0,\;\theta \left(\infty \right)\to 0,\;\varphi \left(\infty \right)\to 0, \end{array}$$ (11)

where primes represent the differentiation with respect to *η*, Re_*a*_ $\left(=\frac{\rho xU}{a}\right)$ and Re_*b*_ $\left(=\frac{\rho x^{n}U^{2-n}}{b}\right)$ denote the local Reynolds numbers, *A* $\left(=\frac{{\text{Re}}_{b}^{2/n+1}}{{\text{Re}}_{a}}\right)$ the material parameter of the Sisko fluid, Pr $\left(=\frac{xU}{\alpha }{\text{Re}}_{b}^{-\frac{2}{n+1}}\right)$ and *γ* $\left(=\frac{h_{f}}{k}x{\text{Re}}_{b}^{-\frac{1}{n+1}}\right)$ represent the generalized Prandtl number and the generalized Biot number, respectively, with *γ* → *∞*, the convective boundary condition reduces to the uniform surface temperature boundary condition, *N*_*b*_ $\left(=\frac{\tau D_{B}(C_{w}-C_{\infty })}{\alpha }\right)$, *N*_*t*_ $\left(=\frac{\tau D_{T}(T_{f}-T_{\infty })}{T_{\infty }\alpha }\right)$ and *Le* $\left(=\frac{\alpha }{D_{B}}\right)$ represent the Brownian motion parameter, thermophoresis parameter and Lewis number, respectively.

The physical quantities of pre-eminent interest are the local skin friction coefficient *C*_*fx*_, the local Nusselt number *Nu*_*x*_ and the Sherwood number *Sh*_*x*_ which can be characterized as:

$$C_{fx}=\frac{{\tau_{xy}|}_{y=0}}{\frac{1}{2}\rho U^{2}},\;Nu_{x}=\frac{x{q_{w}|}_{y=0}}{K\left(T_{f}-T_{\infty }\right)},\;Sh_{x}=\frac{x{j_{w}|}_{y=0}}{D_{B}\left(C_{w}-C_{\infty }\right)},$$ (12)

where *τ*_*xy*_, *q*_*w*_ and *j*_*w*_ are the wall shear stress, heat and mass fluxes, respectively, defined by

$$\tau_{xy}=\left(a+b{\left|\frac{\partial u}{\partial y}\right|}^{n-1}\right)\frac{\partial u}{\partial y},\;q_{w}=-K\left(\frac{\partial T}{\partial y}\right),\;j_{w}=-D_{B}\left(\frac{\partial C}{\partial y}\right).$$ (13)

Using Eqs (6) and (7), the dimensionless parameters can be written in terms of the output of the local-similarity solutions as:

$$\frac{1}{2}{\text{Re}}_{b}^{\frac{1}{n+1}}C_{fx}=Af\prime \prime \left(0\right)-{\left[-f\prime \prime \left(0\right)\right]}^{n},\;{\text{Re}}_{b}^{-\frac{1}{n+1}}Nu_{x}=-\theta \prime \left(0\right),\;{\text{Re}}_{b}^{-\frac{1}{n+1}}Sh_{x}=-\varphi \prime \left(0\right).$$ (14)

## Solution Methodology

### The homotopy analysis method

By employing the HAM [16–18] series solutions are obtained corresponding to the governing non-linear coupled ODEs (8)-(10) and the related boundary conditions (11). Here the initial guesses and operators are chosen as follows:

$$f_{0}(\eta )=1-e^{-\eta },\;\theta_{0}(\eta )=\frac{\gamma e^{-\eta }}{1+\gamma },\;\varphi_{0}(\eta )=e^{-\eta },$$ (15)

$${£}_{f}=\frac{d^{3}}{d\eta^{3}}-\frac{d}{d\eta },\;{£}_{\theta }=\frac{d^{2}}{d\eta^{2}}-1,\;{£}_{\varphi }=\frac{d^{2}}{d\eta^{2}}-1,$$ (16)

for the velocity, temperature and the nanoparticle volume fraction, respectively.

### The numerical method

In general it is very difficult to find the exact analytical solution of non-linear two point boundary value problems (8), (9)and (10) along with boundary conditions (11). Therefore, these problems are solved numerically by the shooting technique [19,20]. The equations are firstly written as a system of seven first order ordinary differential equations and then the corresponding initial value problems are solved by the Runge-Kutta method. The initially guessed values are refined iteratively using the Newtons's method to satisfy boundary condition at infinity. The iterative process is terminated when the absolute error is less than the tolerance 10^−5^.

## Graphical Results and Discussion

The set of coupled Eqs (8) to (10) are highly non-linear equations, subject to boundary conditions (11) constitute a two-point boundary value problem. In general, exact analytic solutions are not possible for the complete set of such equations and hence we used the homotopy analytic method (HAM) for analytic solutions and shooting method for numerical solutions. The obtained results are presented graphically to highlight salient features of the flow, heat transfer and nanoparticle volume fraction transfer characteristics. As a test of the accuracy of the obtained HAM results, a comparison between the present HAM results and those obtained numerically by the shooting technique is presented. Additionally, the accuracy of the present results is confirmed by comparing them with the previously recorded results.

Table 1 presents a comparison of the present results to the previously reported results by Khan and Pop [5], Wang [21] and Gorla and Sidawi [22]. The values of *θ*′(0) have been compared for different values of Prandtl number Pr for the case of Newtonian fluid (i.e., *N*_*b*_ = *N*_*t*_ = 0). It is revealed by the Table 1 that the data produced presently is in good agreement with the previously generated data.

**Table 1:** Comparison of the present results of local Nusselt number –*θ*′(0) for the case of Newtonian fluid with the results of Khan and Pop [5], Wang [21] and Gorla and Sidawi [22].

Pr	Present results	Khan and Pop [5]	Wang [21]	Gorla and Sidawi [22]
0.70	0.45392	0.4539	0.4539	0.5349
2.00	0.91135	0.9113	0.9114	0.9114
7.00	1.89543	1.8954	1.8954	1.8905
20.0	3.35395	3.3539	3.3539	3.3539

**Fig 1:**
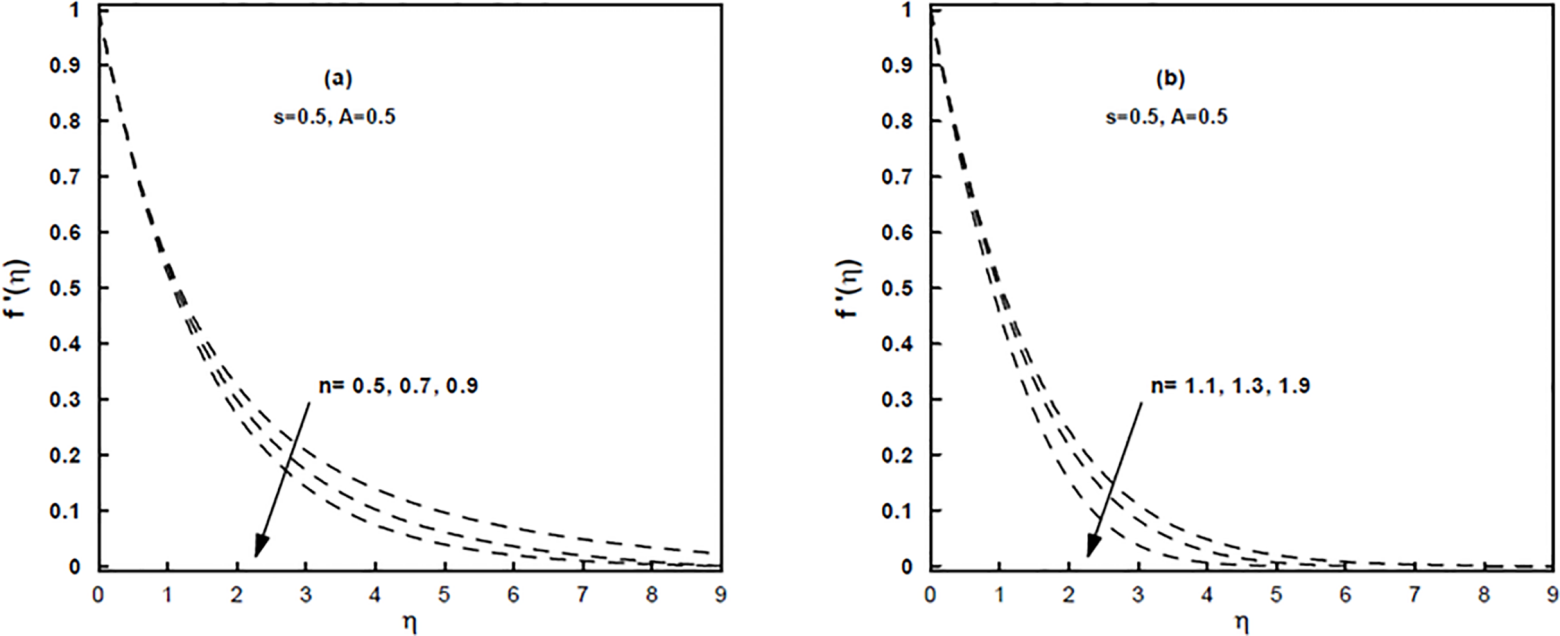
(a,b), The velocity profiles for different values of the power-law index *n*.

**Fig 2:**
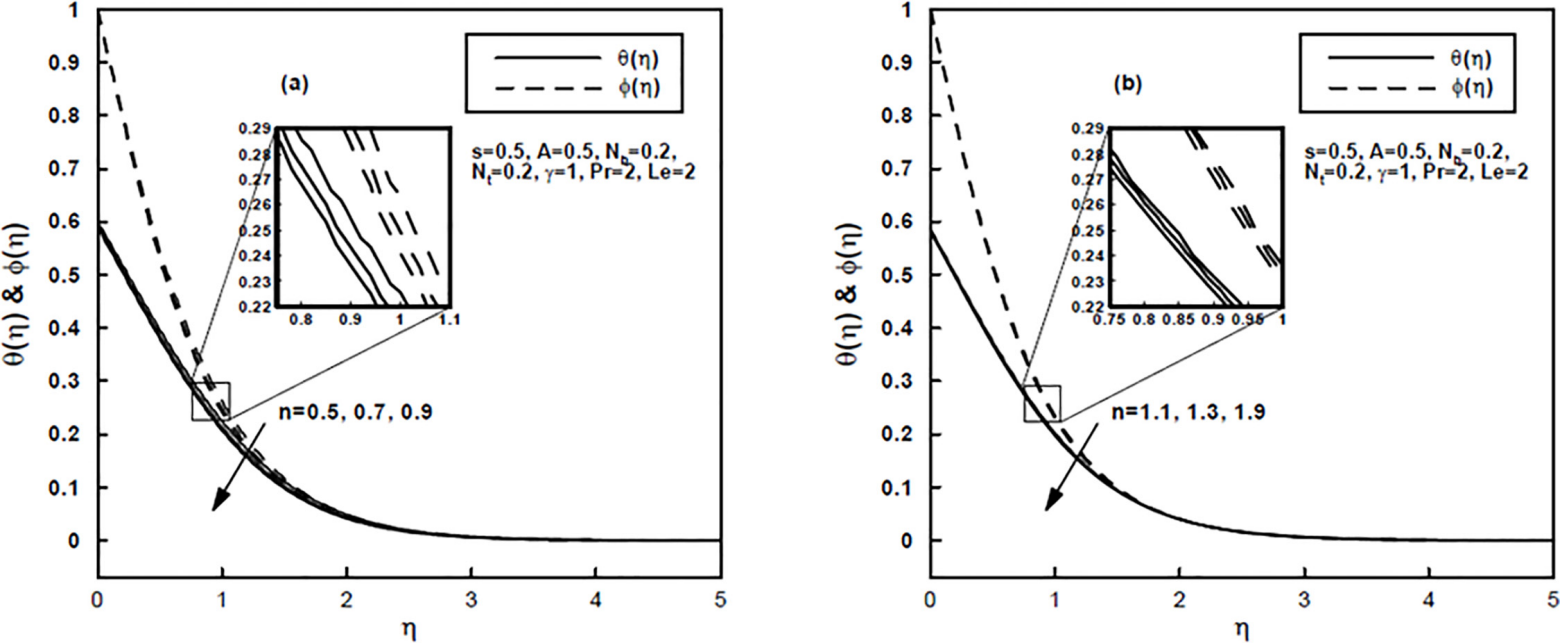
(a,b), The temperature and concentration profiles for different values of the power-law index *n*.

The velocity, temperature and concentration distributions versus the similarity variable *η* for a few values of the power-law index *n* are demonstrated through Figs 1A and 1B and 2A and 2B. It should be noticed that *n* < 1 relates to shear thinning (pseudoplastic) fluids and *n* > 1 relates to shear thickening (dilatant) fluids. It is anticipated by these figures that the velocity, temperature and concentration distributions diminish as *n* increases for both shear thinning and shear thickening fluids. This results in a reduction in the momentum, thermal and concentration boundary layers thickness. Further, it is seen that within the boundary layer the values of *n* affects significantly the velocity distribution but marginally the temperature and concentration distributions for a given *A* and *s*. Note that for *n* < 1, the effective viscosity decreases with increasing shear rate and the viscous effects are transmitted up to a greater distance and thus a reduction in the shear layer (when compared with Newtonian fluid) is a characteristic feature of the Sisko fluid when *n* < 1 (see Fig 1(A)). In the opposite case where the effective viscosity increases with increasing shear rate and consequently, the boundary layer thickness becomes thinner for shear-thickening (1 < *n* < 2) fluids than that of Newtonian (*n* = 1) and shear-thinning (0 < *n* < 1) fluids (see Fig 1(B)).

**Fig 3:**
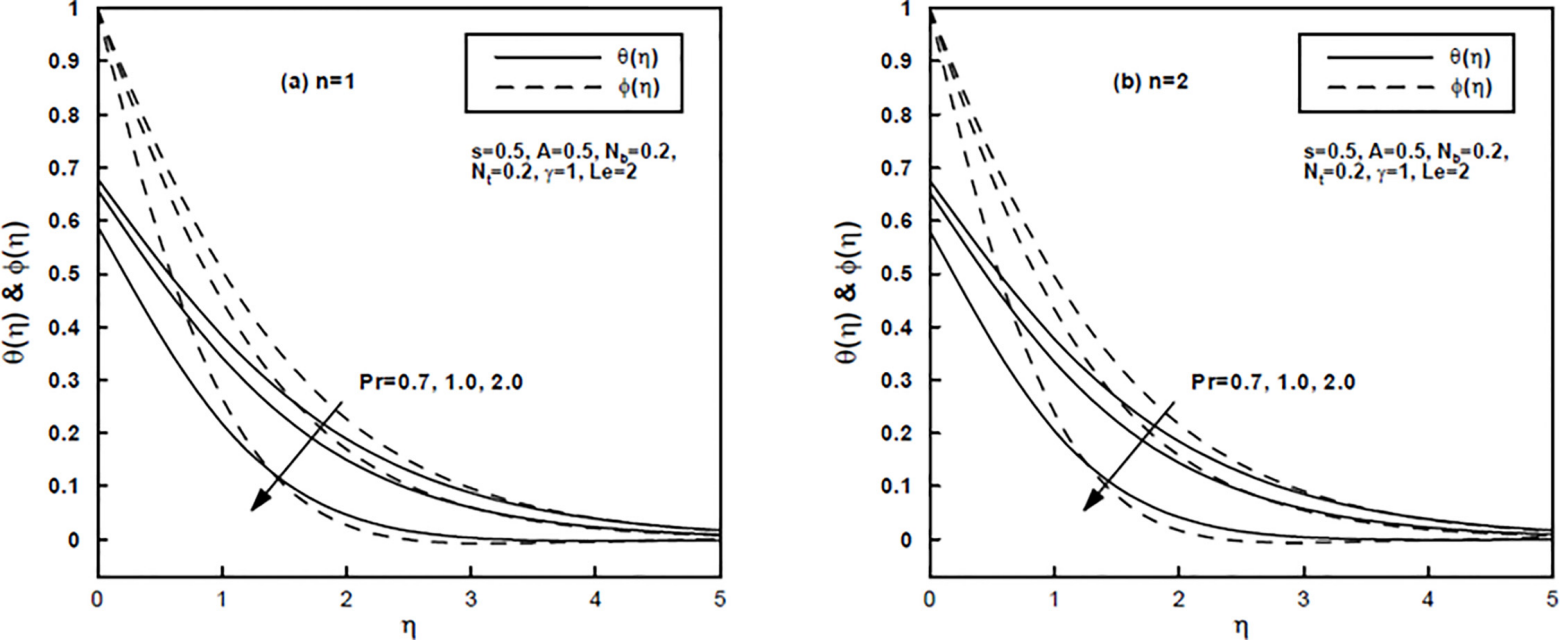
(a,b), The temperature and concentration profiles for different values of the generalized Prandtl number Pr.

**Fig 4:**
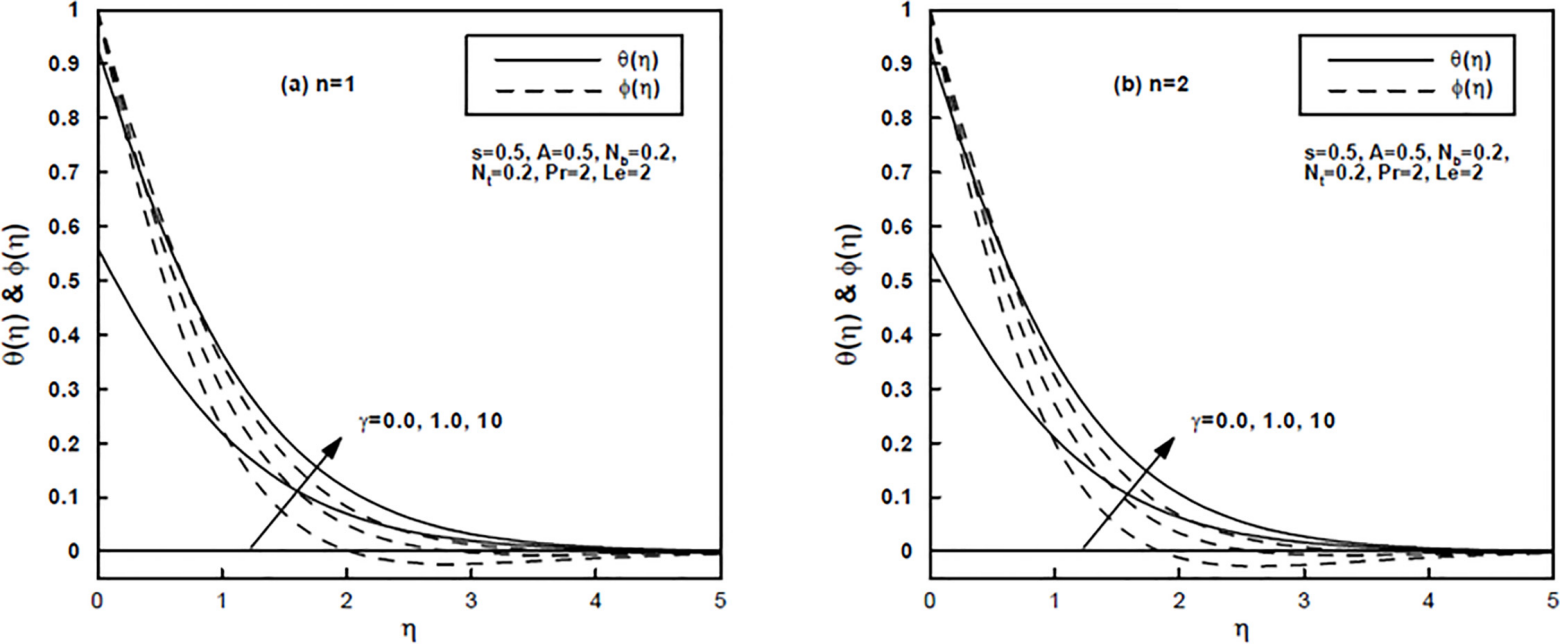
(a,b), The temperature and concentration profiles for different values of the generalized Biot number γ.

The impact of generalized Prandtl number Pr and the generalized Biot number *γ* on the temperature *θ*(*η*) and nanoparticle concentration *φ*(*η*) distributions is shown by Figs 3A and 3B and 4A and 4B for the power-law index *n* = 1 and *n* = 2, respectively. Physically, enhancing the Prandtl number Pr results in a reduction in thermal diffusivity. It can be seen that the temperature and nanoparticle concentration profiles decrease with an increase in the generalized Prandtl number Pr. This further results in reducing the thermal and concentration boundary layers thickness. Note that for high Biot number *γ* convective heating increases and the isothermal surface (i.e., *θ*(0) = 1) is reproduced as *γ* → *∞*. Indeed, a higher generalized Biot number shows elevated internal thermal resistance of the surface than the boundary layer thermal resistance. Therefore, a build in the generalized Biot number increases fluid temperature effectively and Fig 4A and 4B affirm this likewise. In addition, it is noted that an increase in the value of *γ* tends to increase the concentration boundary layer thickness.

**Fig 5:**
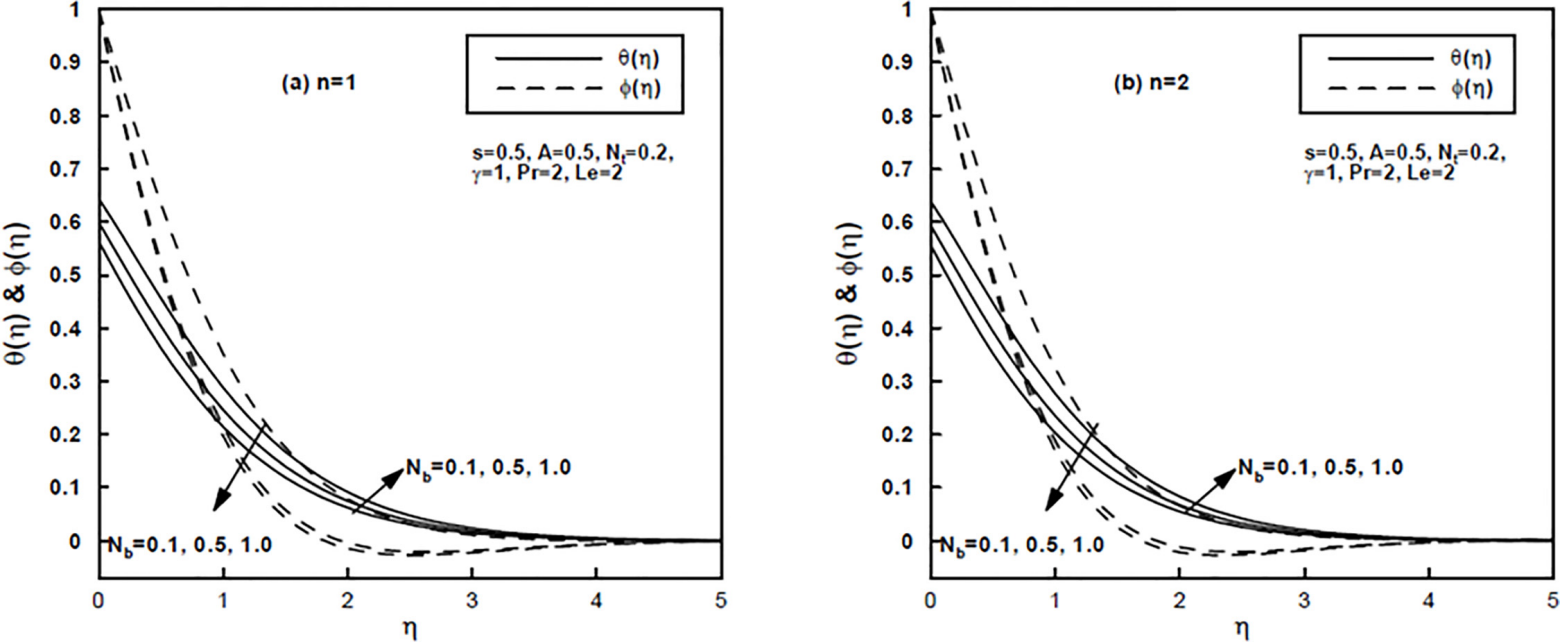
(a,b), The temperature and concentration profiles for different values of the Brownian motion parameter *N*_*b*_.

**Fig 6:**
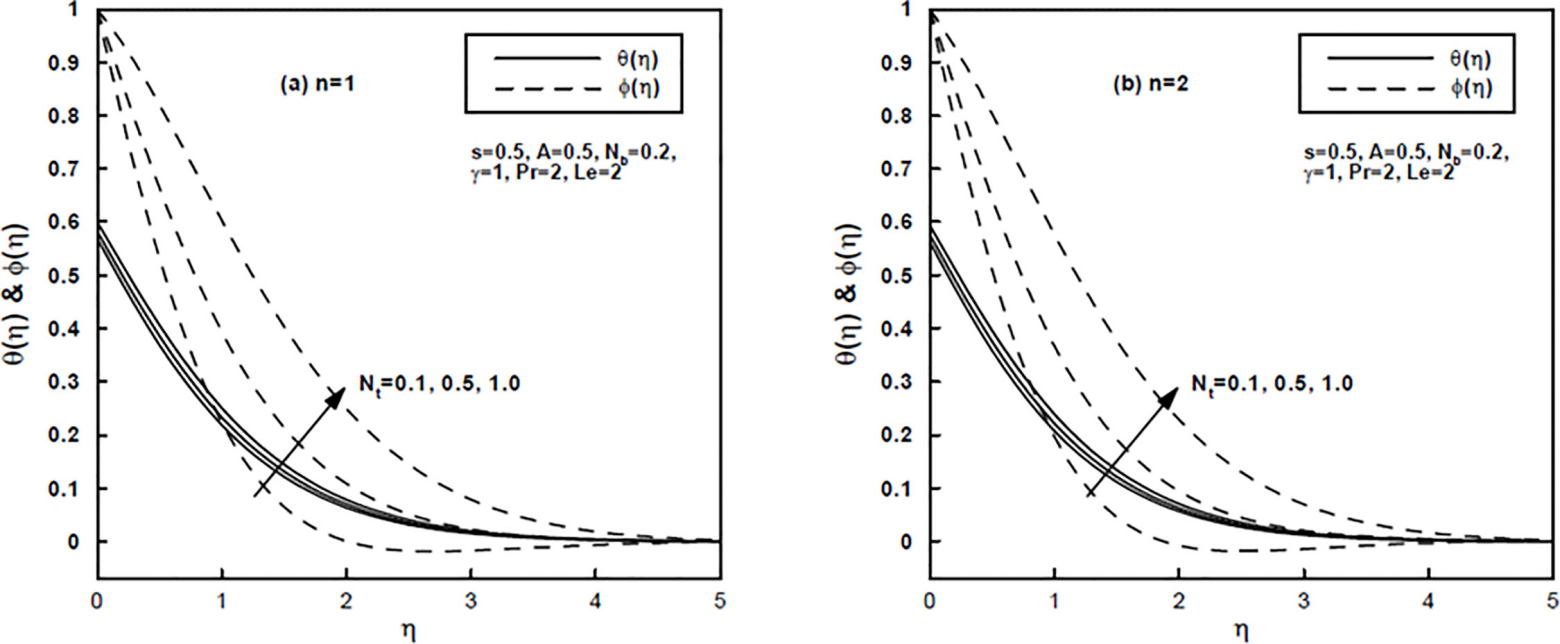
(a,b), The temperature and concentration profiles for different values of the thermophoresis parameter *N*_*t*_.

Figs 5A and 5B and 6A and 6B illustrate the impact of the Brownian motion parameter *N*_*b*_ and the thermophoresis parameter *N*_*t*_ on the temperature and concentration distributions for the power-law index *n* = 1 and *n* = 2, respectively. Physically, the Brownian motion is stronger in case of smaller nanoparticles which corresponds to the larger *N*_*b*_ and converse is the situation for smaller values of *N*_*b*_. In thermal conduction nanoparticle's motion plays a pivotal role. Due to the intensified chaotic motion of the nanoparticles (i.e., for larger *N*_*b*_) the kinetic energy of the particles is enhanced which as a result enhances the temperature of the nanofluid. Hence increasing *N*_*b*_ firmly raises temperature values throughout the regime as demonstrated in Fig 5A and 5B. Away from the surface the larger values of *N*_*b*_ stifle the diffusion of the nanoparticles in the fluid regime which reduces the concentration distribution. For higher values of *N*_*b*_ larger thermal boundary layer thickness is formed though larger concentration boundary layer thickness is produced for smaller values of *N*_*b*_. On the other hand, in nanofluid flow the thermophoresis parameter *N*_*t*_ plays a pivotal role in examining the temperature and nanoparticle concentration distributions. Due to the larger values of *N*_*t*_ the thermophoretic forces are produced. These forces have the tendency to migrate the nanoparticles in the reverse direction of temperature gradient (i.e., from hot to cold) which causes a non-uniform nanoparticle distribution. Consequently, the increasing values of *N*_*t*_ corresponds to an increase in the temperature and nanoparticle concentration distributions as shown in Fig 6A and 6B.

**Fig 7:**
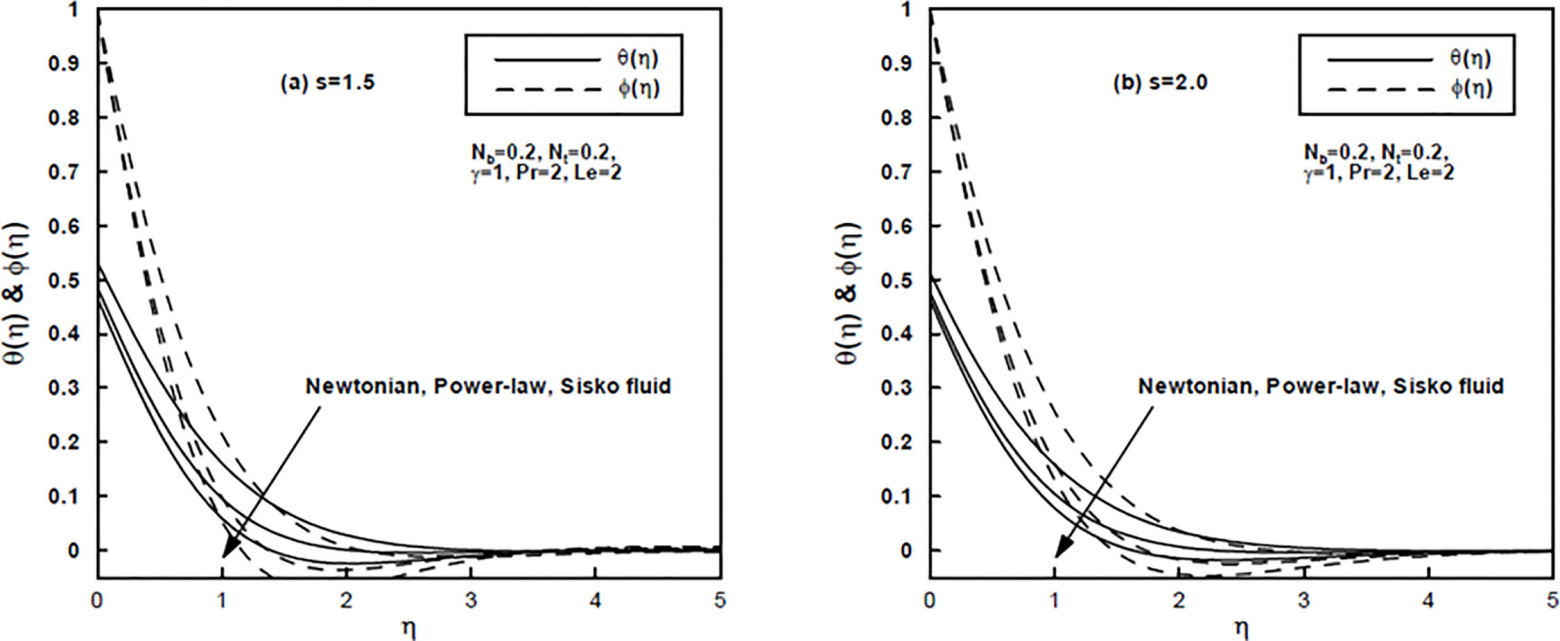
(a,b), A comparison of the temperature and concentration profiles for the Newtonian, power-law and Sisko fluids.

Fig 7A and 7B compare the temperature and concentration profiles of the Newtonian (*A* = 0, *n* = 1) and the power-law (*A* = 0, *n* ≠ 1) fluid with those of the Sisko (*A* ≠ 0, *n* ≠ 1) fluid for the stretching parameter *s* = 1.5 and *s* = 2.0, respectively. Straightforwardly, it can be seen from these figures that the thermal and nanoparticle concentration profiles are larger for the Newtonian and power-law fluids as compared to those of the Sisko fluid. Additionally, the temperature and concentration boundary layers are highly dependent on the stretching parameter *s* and they diminishes for stronger *s*.

**Fig 8:**
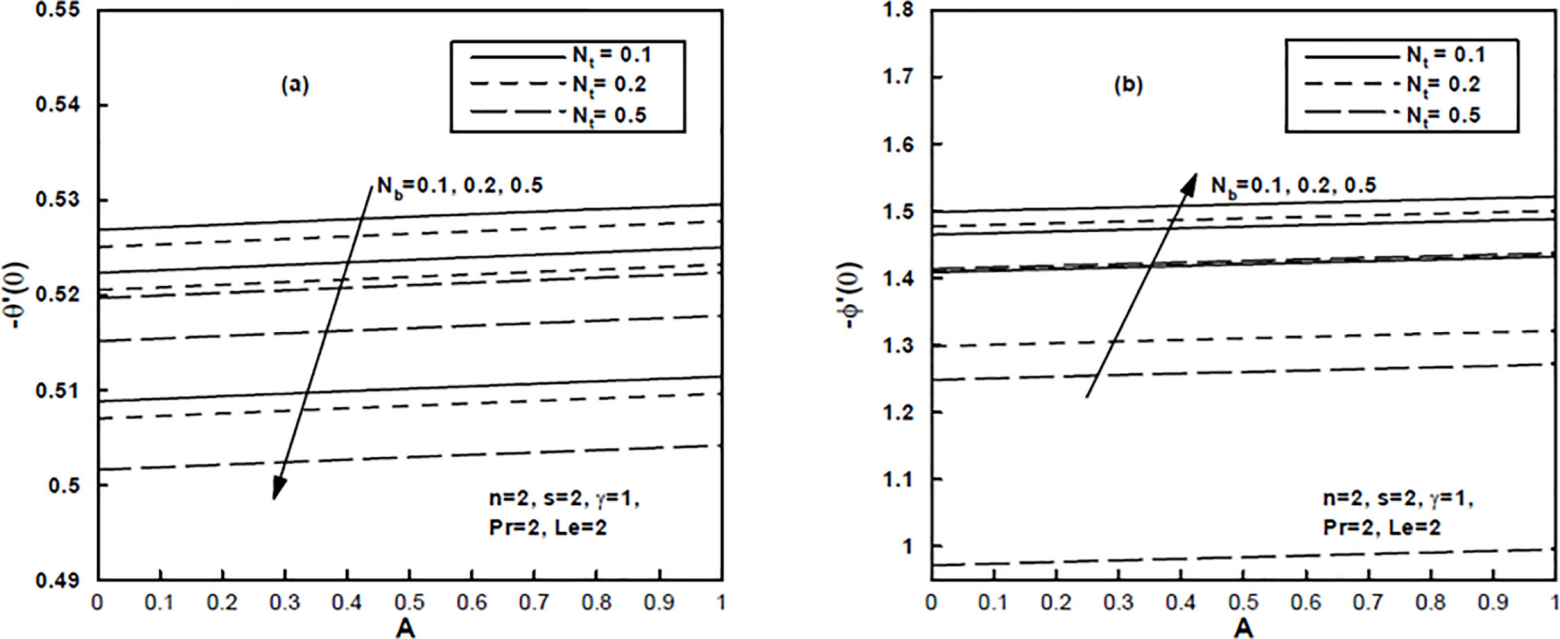
(a,b), Influence of the Brownian motion parameter *N*_*b*_ and the thermophoresis parameter *N*_*t*_ on the local Nusselt number and the local Sherwood number.

The effects of the thermophoresis parameter *N*_*t*_ and the Brownian motion parameter *N*_*b*_ on local Nusselt number ${\text{Re}}_{b}^{-\frac{1}{n+1}}Nu_{x}$ and local Sherwood number ${\text{Re}}_{b}^{-\frac{1}{n+1}}Sh_{x}$ are shown in Fig 8A and 8B. These figures indicate a decrease in the values of both local Nusselt number and local Sherwood number with the increase in thermophoresis parameter *N*_*t*_. Further, it can be seen that the effect of the Brownian motion parameter *N*_*b*_ is to decrease the local Nusselt number but its effect is quite opposite on the local Sherwood number.

These results evidently meet with the data provided in Table 2. Additionally, from Table 2 it is evident that the magnitude of heat transfer rate *θ*′(0) increases with the increase in the value of the material parameter *A*, stretching parameter *s*, generalized Prandtl number Pr and generalized Biot number *γ* but an opposite behavior can be seen for the Lewis number *Le*. Also, an increasing magnitude of rate of concentration *φ*′(*η*) is straightforwardly observed for the larger material parameter *A*, stretching parameter *s*, generalized Prandtl number Pr and Lewis number *Le* while it is decreasing for the generalized Biot number *γ*.

**Table 2:** Variation of the local Nusselt number ${\text{Re}}_{b}^{-\frac{1}{n+1}}Nu_{x}$ and local Sherwood number ${\text{Re}}_{b}^{-\frac{1}{n+1}}Sh_{x}$ for different values of emerging physical parameters.

*A*	*s*	Pr	*N*_*b*_	*N*_*t*_	*γ*	*Le*	$-{\text{Re}}_{b}^{-\frac{1}{n+1}}Nu_{x}$		$-{\text{Re}}_{b}^{-\frac{1}{n+1}}Sh_{x}$	
							*n* = 1	*n* = 2	*n* = 1	*n* = 2
0.0	0.5	1.0	0.1	0.1	0.1	1.5	0.082857	0.074467	0.622412	0.715660
0.5							0.083721	0.083772	0.658103	0.668808
1.0							0.084201	0.084494	0.679343	0.700421
0.5	0.5	0.1	0.1	0.1	0.1	1.5	0.083721	0.083772	0.658103	0.668808
	1.0						0.085352	0.086843	0.752096	0.864615
	2.0						0.087524	0.089886	0.915241	1.174931
0.5	0.5	0.7	0.1	0.1	0.1	1.5	0.080291	0.080044	0.518304	0.517209
		1.0					0.083721	0.083772	0.658103	0.668808
		1.3					0.085841	0.086024	0.777992	0.798376
0.5	0.5	1.0	0.1	0.1	0.1	1.5	0.083721	0.083772	0.658103	0.668808
			0.5				0.080131	0.080162	0.696729	0.710328
			1.0				0.074472	0.074467	0.701720	0.715660
0.5	0.5	1.0	0.1	0.1	0.1	1.5	0.083721	0.083772	0.658103	0.668808
				0.5			0.083402	0.083465	0.475450	0.470141
				1.0			0.082981	0.083059	0.253868	0.227181
0.5	0.5	1.0		0.1	0.1	1.5	0.083721	0.083772	0.658103	0.668808
					0.5		0.252072	0.252584	0.565438	0.567982
					1.0		0.335722	0.336674	0.519698	0.517973
0.5	0.5	1.0	0.1	0.1	0.1	1.2	0.083783	0.083838	0.564987	0.568379
						1.5	0.083721	0.083772	0.658103	0.668808
						2.0	0.083645	0.083693	0.793965	0.814913

**Fig 9:**
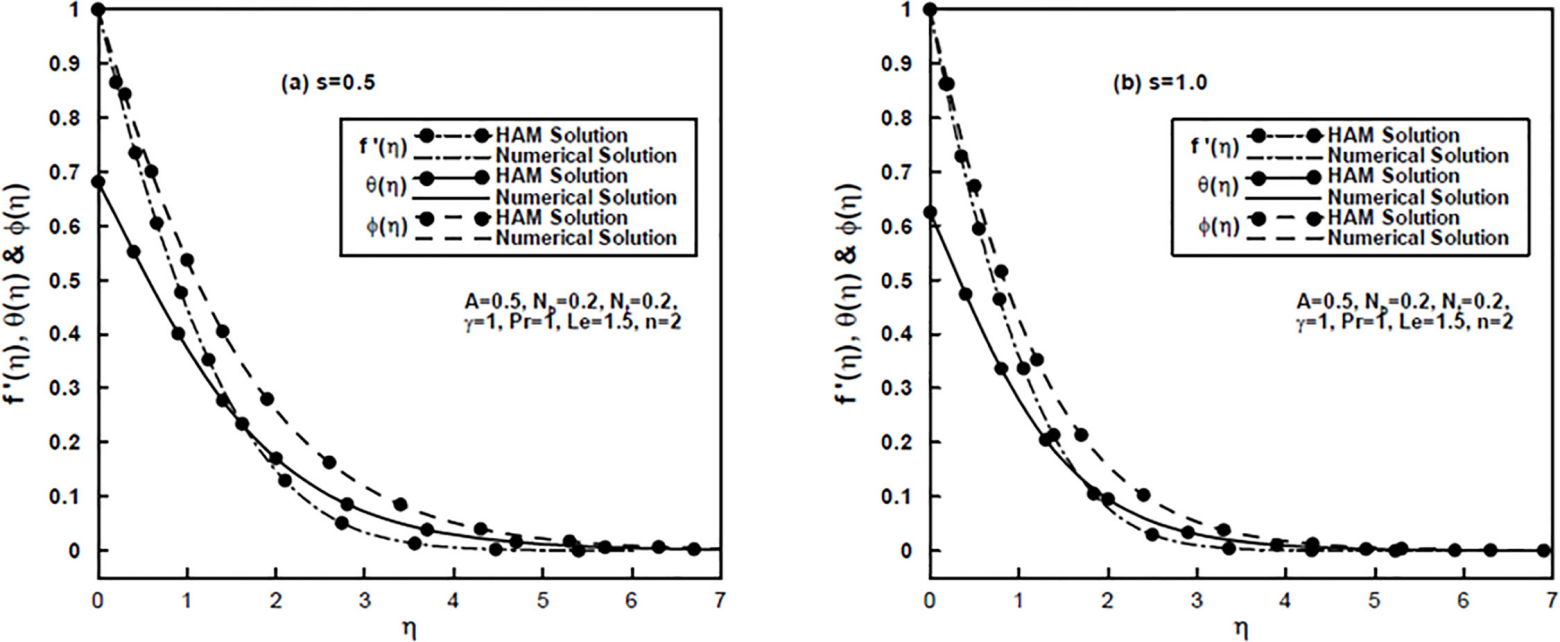
(a,b), A comparison of HAM solutions and numerical solutions for the velocity, temperature and concentration profiles.

In Fig 9A and 9B a comparison is made between the results obtained by homotopy analysis method (HAM) and solutions obtained by numerical method. Interestingly, it is found that both the results show good agreement which validates the HAM results.

## Summary and Conclusions

The influence of forced convection to Sisko nanofluid over a stretching sheet was intended to investigate in this study. Thermal convective boundary conditions were also considered. This study was conducted under the consideration of the model proposed by Buongiorno [4]. The three coupled nonlinear governing equations were transformed to the corresponding ordinary differential equations by using appropriate transformations. These ordinary differential equations were further solved analytically (by homotopy analysis method) and numerically (by shooting technique) to obtain the results (i.e., velocity, temperature and concentration profiles). To analyze the behavior of these results under the influence of different physical parameters graphs and tables were constructed.

From these graphs the behavior of nanofluid temperature distribution and thermal boundary layer thickness could be noticed as monotonically increasing for the thermophoresis parameter *N*_*t*_, Brownian motion parameter *N*_*b*_ and generalized Biot number *γ* while it was decreasing for the material parameter *A*, generalized Prandtl number Pr, power-law index *n* and stretching parameter *s*. Also, the concentration profile was notice to increase for *N*_*t*_ and *γ* whereas it was decreased for the material parameter *A*, generalized Prandtl number Pr, power-law index *n*, stretching parameter *s* and Brownian motion parameter *N*_*b*_. Additionally, from the Table 2 it appeared that a rise in the values of the material parameter *A*, generalized Prandtl number Pr and generalized Biot number *γ* resulted in reducing the rate of heat transfer whereas the effect was reverse for Brownian motion parameter *N*_*b*_, thermophoresis parameter *N*_*t*_ and Lewis number *Le*. Further, the mass transfer rate enhanced for larger values of material parameter *A*, Prandtl number Pr, Brownian motion parameter *N*_*b*_ and Lewis number *Le* but diminished for rising values of *N*_*t*_ and *γ*.

## References

[1] Choi SUS (1995) Enhancing thermal conductivity of fluids with nanoparticles; Development and applications of non-Newtonian flows. DA Siginer and HP Wang Eds ASME MD 231 99–105.

[2] Kwak K, Kim C (2005) Viscosity and thermal conductivity of copper nanofluid dispersed in ethylene glycol. Korea Aust Rheol J 17 35–40.

[3] Masuda H, Ebata A, Teramae K, Hishinuma N (1993) Alteration of thermal conductivity and viscosity of liquid by dispersing ultra-fine particles. Netsu Bussei 7 227–233.

[4] Buongiorno J (2006) Convective transport in nanofluids. J Heat Transf 128 (3) 240–250.

[5] Khan WA, Pop I (2010) Boundary-layer flow of a nanofluid past a stretching sheet. Int J Heat Mass Transf 53 2477–2483.

[6] Rahman MM, Rosca AV, Pop I (2014) Boundary layer flow of a nanofluid past a permeable exponentially shrinking/stretching surface with second order slip using Buongiorno's model. Int J Heat Mass Transf 77 1133–1143.

[7] Rohni AM, Ahmad S, Ismail AIM, Pop I (2013) Flow and heat transfer over an unsteady shrinking sheet with suction in a nanofluid using Buongiorno's model. Int Commun Heat Mass Transf 43 75–80.

[8] Rosca NC, Pop I (2014) Unsteady boundary layer flow of a nanofluid past a moving surface in an external uniform free stream using Buongiorno's model. Comput Fluids 95 49–55.

[9] Zaimi K, Ishak A, Pop I (2014) Unsteady flow due to a contracting cylinder in a nanofluid using Buongiorno's model. Int J Heat Mass Transf 68 509–513.

[10] Malvandi A, Ganji DD (2014) Brownian motion and thermophoresis effects on slip flow of alumina/water nanofluid inside a circular microchannel in the presence of a magnetic field. Int J Thermal Sci 84 196–206.

[11] Khan WA, Aziz A (2011) Double-diffusive natural convective boundary layer flow in a porous medium saturated with a nanofluid over a vertical plate: Prescribed surface heat, solute and nanoparticle fluxes. Int J Thermal Sci 50 (11) 2154–2160.

[12] Hady FM, Ibrahim FS, Abdel-Gaied SM, Eid MR (2012) Radiation effect on viscous flow of a nanofluid and heat transfer over a nonlinearly stretching sheet. Nanoscale Research Letters 7:229 10.1186/1556-276X-7-229 22520273 PMC3349597

[13] Hady FM, Eid MR, Ahmed MA (2014) A nanofluid flow in a non-linear stretching surface saturated in a porous medium with yield stress effect. Appl Math Inf Sci Lett 2 2 43–51.

[14] Sisko AW (1958) The flow of lubricating greases. Industrial and Engineering Chemistry Research 50(12) 1789–1792.

[15] Sanjayanand E, Khan SK (2006) On heat and mass transfer in a viscoelastic boundary layer flow over an exponentially stretching sheet. Int J Thermal Sci 45 819–828. 16863518

[16] Hayat T, Shafiq A, Alsaedi A (2014) Effect of joule heating and thermal radiation in flow of third grade fluid over radiative surface. PLoS ONE 9(1):e83153 10.1371/journal.pone.0083153 24454694 PMC3893084

[17] Hayat T, Shafiq A, Alsaedi A, Awais M (2013) MHD axisymmetric flow of third grade fluid between stretching sheets with heat transfer. Comput Fluids 86 103–108.

[18] Khan WA, Khan M, Malik R (2014) Three-dimensional flow of an Oldroyd-B nanofluid towards stretching surface with heat generation/absorption. PLoS ONE 9(8): e105107 10.1371/journal.pone.0105107 25170945 PMC4149422

[19] Malik R, Khan M, Munir A, Khan WA (2014) Flow and Heat Transfer in Sisko Fluid with Convective Boundary Condition. PLoS ONE 9(10): e107989 10.1371/journal.pone.0107989 25285822 PMC4186795

[20] Munir A, Shahzad A, Khan M (2014) Forced convective heat transfer in boundary layer flow of sisko fluid over a nonlinear stretching sheet. PLoS ONE 9(6): e100056 10.1371/journal.pone.0100056 24949738 PMC4064994

[21] Wang CY (1989) Free convection on a vertical stretching surface. J Appl Math Mech (ZAMM) 69 418–420.

[22] Gorla RSR, Sidawi I (1994) Free convection on a vertical stretching surface with suction and blowing. Appl Sci Res 52 247–257.

